# Lack of MERS Coronavirus Neutralizing Antibodies in Humans, Eastern Province, Saudi Arabia

**DOI:** 10.3201/eid1912.130701

**Published:** 2013-12

**Authors:** Stefanie Gierer, Heike Hofmann-Winkler, Waleed H. Albuali, Stephanie Bertram, Abdullah M. Al-Rubaish, Abdullah A. Yousef, Awatif N. Al-Nafaie, Amein K. Al-Ali, Obeid E. Obeid, Khaled R. Alkharsah, Stefan Pöhlmann

**Affiliations:** German Primate Center, Göttingen, Germany (S. Gierer, H. Hofmann-Winkler, S. Bertram, S. Pöhlmann);; University of Dammam, Dammam, Saudi Arabia (W.H. Albuali, A.M. Al-Rubaish, A.A. Yousef, A.N. Al-Nafaie, A.K. Al-Ali, O.E. Obeid, K.R. Alkharsah).

**Keywords:** MERS coronavirus, neutralizing antibodies, Saudi Arabia, spike, pseudotyping, MERS-CoV, viruses, coronavirus, Middle East respiratory syndrome coronavirus, seroprevalence, serum, plasma, respiratory infections

## Abstract

We used a lentiviral vector bearing the viral spike protein to detect neutralizing antibodies against Middle East respiratory syndrome coronavirus (MERS-CoV) in persons from the Eastern Province of Saudi Arabia. None of the 268 samples tested displayed neutralizing activity, which suggests that MERS-CoV infections in humans are infrequent in this province.

The emergence of the Middle East respiratory syndrome coronavirus (MERS-CoV, formerly termed EMC coronavirus [*1*]) could pose a serious threat to public health (*2*). As of September 2013, a total of 108 laboratory-confirmed infections (with 50 deaths) caused by MERS-CoV have been reported to the World Health Organization (WHO), most from Saudi Arabia (*3*), but data are limited on MERS-CoV seroprevalence in humans (*4*).

We recently developed a lentiviral vector system to study host cell entry mediated by the spike protein of MERS-CoV (MERS-S) (*5*). This system mimics key aspects of MERS-CoV cellular entry and enables sensitive and quantitative detection of neutralizing antibodies, which are known to be generated in infected patients (*5*). We used this system to determine the presence of MERS-CoV neutralizing antibodies in serum and plasma samples obtained from patients at King Fahd Hospital of the University in Alkhobar, Saudi Arabia. The hospital is a referral hospital that serves the Eastern Province of Saudi Arabia, including the Dammam and Alhasa governorates, from which several MERS cases were reported, according to the Ministry of Health of Saudi Arabia and a recent study (*6*); no MERS patients were seen at King Fahd Hospital. Blood collection for this study was approved by the University of Dammam ethics committee, and informed, written consent for participation was received for all study participants.

## The Study

Two collections of patient samples were analyzed. The first collection consisted of 158 serum samples taken from children hospitalized for lower respiratory tract infections during May 2010–May 2011. The samples came from 77 female and 81 male patients with a median age of 11.6 months (range 7.3 months to 9 years). The second sample collection consisted of 110 plasma samples from men with a median age of 28 years (range 19–52 years) who donated blood at the hospital during December 2012.

**Figure 1:**
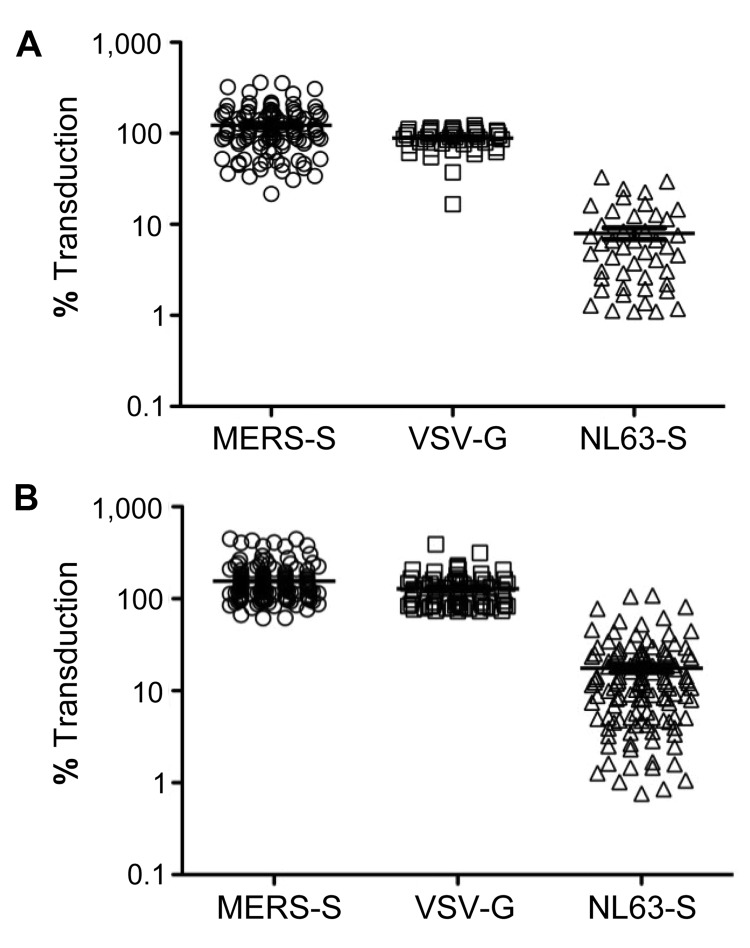
Neutralizing activity of serum and plasma samples obtained from patients at King Fahd Hospital of the University in Alkhobar, Saudi Arabia. A) Lentiviral vectors encoding luciferase and bearing the indicated viral glycoproteins were incubated with 1:20 dilutions of plasma from healthy adults, obtained during December 2012, and then added to target cells. Transduction efficiency was measured by quantification of luciferase activities in cell lysates and is shown relative to transduction of cells in the absence of serum, which was set at 100%. All 110 plasma samples available were tested for neutralization of Middle East respiratory syndrome coronavirus spike protein (MERS-S)–dependent transduction; subsets were also tested for neutralization of transduction driven by the G protein of vesicular stomatitis virus (VSV-G) (46/110) and the S protein of human coronavirus NL63 (NL63-S) (46/110). B) Analysis conducted as described for panel A using 158 serum samples from children with lower respiratory tract infections, obtained during May 2010–May 2011. All samples were analyzed for neutralization of MERS-S–mediated transduction; subsets were also tested for neutralization of transduction driven by VSV-G (76/158) and NL63-S (123/158). Horizontal lines indicate mean ±SEM.

**Figure 2:**
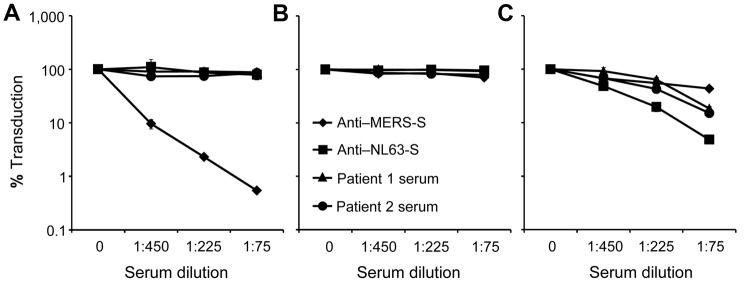
Analysis of serum samples with known neutralizing activity. Neutralization of transduction driven by the Middle East respiratory syndrome coronavirus spike protein (MERS-S) (A), G protein of vesicular stomatitis virus (B), and S protein of human coronavirus NL63 (NL63-S) (C) were determined as described for Figure 1, except that serum with known reactivity to MERS-S and NL63-S and serum from 2 patients at King Fahd Hospital of the University in Alkhobar, Saudi Arabia, that neutralized NL63-S–mediated transduction (Figure 1, panel A) were analyzed. Transduction of target cells in the absence of serum was set at 100%.

Analysis of MERS-S–driven transduction of target cells revealed that none of the samples investigated contained neutralizing antibodies against MERS-S (Figure 1). As a control, a subset of the samples was analyzed for inhibition of cellular entry mediated by the G protein of vesicular stomatitis virus (VSV-G), an animal virus that does not circulate in Saudi Arabia, and the spike protein of the human coronavirus NL63 (NL63-S), a globally circulating coronavirus. None of the samples robustly inhibited VSV-G–dependent entry, whereas most samples markedly reduced entry-driven by NL63-S (Figure 1), as expected (*7*). Experiments using serum samples of known neutralizing capacity confirmed that our neutralization experiments were sensitive and specific (Figure 2). Thus, serum samples obtained from a patient infected with MERS-CoV potently inhibited MERS-S but not VSV-G– or NL63-S–driven entry, whereas the reverse observation was made with serum samples reactive against NL63-S (Figure 2). In sum, none of the samples from children with respiratory infections and none of the samples from healthy adult men showed detectable amounts of MERS-S–neutralizing antibodies, but most neutralized NL63-S–driven host cell entry.

## Conclusions

Our results suggest that the estimated MERS-CoV seroprevalence in the area served by King Fahd Hospital was <2.3% in children during 2010–2011 and <3.3% in male adults in 2012 (upper limits of the 95% CIs for 0/158 and 0/110, respectively, by Fisher exact test). Our analysis of samples from children might have underestimated seroprevalence because if they were hospitalized for MERS-CoV infection, a virus-specific antibody response might have developed after sample collection. Moreover, although infection of young children has been reported (*8*), the average age of MERS patients is 50 years. Our findings using samples from adult men argue against the extensive spread of MERS-CoV within this group in the Eastern Province of Saudi Arabia during 2012, which is noteworthy given recent reports of asymptomatic MERS-CoV infections (*9**,**10*). 

We cannot rule out that other diagnostic methods that are not limited to detection of neutralizing antibodies might have identified positive samples in our collection. Future analyses are required to determine MERS-CoV seroprevalence in larger patient collectives and in animal species, such as dromedary camels, that could transmit the virus to humans (*4**,**11*).
